# Effect of Zinc Oxide Nanoparticles on Capsular Gene Expression in *Klebsiella pneumoniae* Isolated from Clinical Samples

**DOI:** 10.3390/biomimetics7040180

**Published:** 2022-10-27

**Authors:** Nuha B. Kudaer, Mohseen H. Risan, Emad Yousif, Mohammed Kadhom, Rasha Raheem, Israa Salman

**Affiliations:** 1 College of Biotechnology, Al-Nahrain University, Baghdad 64021, Iraq; 2 Department of Chemistry, College of Science, Al-Nahrain University, Baghdad 64021, Iraq; 3 Department of Environmental Science, College of Energy and Environmental Science, Al-Karkh University of Science, Baghdad 10081, Iraq; 4 Alkarkh Health Directorate, Baghdad 10011, Iraq; 5 College of Science, Al-Nahrain University, Baghdad 64021, Iraq

**Keywords:** *Klebsiella pneumoniae*, nanoparticles, zinc oxide, gene expression, antibacterial

## Abstract

*Klebsiella pneumoniae* is an opportunistic pathogen with various virulence factors that give it the capability to invade a host. Nevertheless, the treatment of bacterial infection is gradually complicated as the bacteria can develop resistance to antimicrobial agents. As nanotechnology is a prosperous field for researchers, we employed zinc oxide (ZnO) nanoparticles (NPs) on isolates of *Klebsiella pneumoniae*. Here, we studied the effect of three NP concentrations—0.25, 0.50, and 0.75 mM—on the gene expression of *Klebsiella pneumoniae* capsules in isolates collected from different samples. After conducting an anti-bacterial test, the highest nine types of bacteria that resisted the antibacterial agent were chosen for further examination. The gene expression of four genes responsible for capsule manufacturing, namely *magA*, *k2A*, *rmpA*, and *kfu*, were investigated. When the NP concentration was 0.25 mM, the lowest efficiency was obtained. However, when the concentration increased to 0.50 mM, a noticeable effect on gene expression was detected; consequently, at a concentration of 0.75 Mm, the highest impact was achieved and the gene expression was stopped.

## 1. Introduction

*Klebsiella pneumoniae* is a broadly dispersed pathogen in healthy individuals’ respiratory, urinary, and gastrointestinal tracts. It is a frequently acquired bacteria in hospitals that causes severe respiratory infections, including pneumonia and other opportunistic illnesses. This type of bacteria can also cause primarily nosocomial and urinary tract infections. However, wound infection, abscesses, sepsis, inflammation, and diarrhea that are associated with *K. pneumoniae* infections have significant death rates if incorrectly managed. Hence, managing *K. pneumoniae* infections is challenging [1]. *K. pneumoniae* has a variety of virulence characteristics that enable it to invade the host, including siderophores, fimbriae, serum resistance, lipopolysaccharide, capsular polysaccharide, and enterotoxin and urea production [2]. However, the fact that this bacteria has resistivity to a vast range of medicines, particularly -lactam antibiotics, is the main reason for its pathogenicity. This is a result of repeated hospital-acquired infections that ultimately forced scientists to consider alternative types of treatments [3]. Isolates of *K. pneumoniae* produce distinct capsular structures made of intricate acidic polysaccharides. There are significant variations in virulence among the serotypes. The K1 serotype, followed by K2, is thought to be among the most pathogenic since they are connected to extremely invasive illnesses [4]. The serotype-specific genes are largely conserved on each side between the serotypes that make up the capsular polysaccharide (cps) gene cluster synthesis [5]. Using particular primers, PCR was carried out to assess the spread of the genes of regulator of mucoid phenotype A (rmpA), mucoviscosity-associated gene A (*magA*), iron uptake system (*kfu*), K2 capsule-associated gene A (*k2A*), and regulator of mucoid phenotype A (*rmpA*) in *K. pneumoniae*. Except for *rmpA*, which is found in plasmids, all of the target genes are chromosomal [6]. The tendency of bacteria to generate resistance against antimicrobial agents results in a serious challenge to treating bacterial illnesses. To control microbial infection, several novel tactics have been used.

Metal oxide NPs are a group of materials that have recently become highly recognized thanks to their potential in scientific and medical applications. Today’s investigations showed the strong antibacterial potential of carefully designed metal oxide NPs [7]. The antibacterial compositions based on nanoparticles could be used as effective bactericidal substances in contemporary medicine [8]. Because of its distinct chemical, electrical, and optical functions, ZnO, one of many metal oxides, has recently drawn particular attention in medical applications [9]. It was reported that ZnO NPs demonstrated potent antibacterial activity versus many harmful microorganisms [10]. There are two probable pathways connected to bacterial inhibition when treated with ZnO: the eventual penetration of the cell’s envelope and bacterial membrane disruption [11,12]. 

On the subject of investigating ZnO NPs and antibacterial activity, however, ongoing research is conducted to explain this impact. This method of treatment could decrease the significant harm that results in the patient’s mortality in the absence of exact and prior detection due to the frequent prevalence of Klebsiella spp. and its high virulence. The aim of the recent study is to investigate the effect of ZnO NPs on the capsule gene expression that causes *Klebsiella pneumoniae*.

## 2. Materials and Methods

### 2.1. ZnO NP Synthesis

ZnO NPs were prepared via the direct precipitation approach, employing KOH and Zn(NO₃)₂ as precursors. They were prepared and characterized as mentioned in our previous work [13]. It is good to mention that the NP size was 27.34, as measured by atomic force microscopy (AFM).

### 2.2. Bacteria Isolation

About 30 strains of *Klebsiella pneumoniae* were isolated from different hospitals located in Baghdad/Iraq, namely: Al-kindy, Ibn Al-baladi, Al-Wasity, Central Children, and the educational lab at Medical City. The strains were collected using sterile containers and the transport swabs were dampened in normal saline. After being cultured on blood agar and MacConkey plates, all isolates were obtained from the clinical samples. These plates underwent an overnight aerobic incubation at 37 °C before being examined for bacterial growth. In order to distinguish the lactose (pink) and non-lactose (colorless) fermenting bacteria, the pinky and mucous colonies were sub-cultured on MacConkey agar, whereas they appeared pale on blood agar and produced gamma-hemolysis results [14]. The colony morphology, staining reactions, and biochemical tests were used to identify probable isolates, and the outcomes were verified via the API 20E and VITEK 2 systems.

### 2.3. The Coculture of ZnO NPs and Klebsiella Pneumoniae

Concentrations of 0.25, 0.50, and 0.75 mM of ZnO NPs were set into a 5 mL sterile nutrient broth of pH = 7.4. Then, around 0.1 mL of the standardized bacterial cell suspension was filled and incubated for a whole day at 37 °C.

### 2.4. Gene Expression

Estimating and analyzing the levels of gene expression for one gene or more relies on the resulting RNA/miRNA concentration after the conversion to cDNA. The processes of total RNA purification, qPCR amplification, and data analysis were included.

#### 2.4.1. Total RNA Purification

RNA was isolated from specimens in accordance with the MagPurix^®^ (New Taipei City, Taiwan) protocol and as illustrated in the following steps:

- A—Preparation of the sample

The cell culture was obtained by centrifuging at 1.000× *g* at 4 °C for 5 min. Then, the supernatants were completely removed and resuspended in cell pellets of 220 μL at 4 °C RL lysis buffer. A vortex mixing for 10 s was applied and 200 μL of the sample was taken to the sample tube.

- B—Purification protocol, MagPurix^®^ series

An appropriate volume of sample was transferred into sample tubes. The protocol barcodes were scanned to select the purification protocol, sample, and elute. Then, the experiment was started by pressing ENTER. The instrument runs the protocol program automatically until the whole process is completed. The purified nucleic acids were stored at 4 °C (short-term, less than 10 days) before performing downstream analysis.

#### 2.4.2. Synthesis of cDNA from RNA Template Protocol

The reaction components (5 μg of template RNA and up to 20 μL of RNase-free water) were added to the RT FDmix (Hexamer). Then, the thermal cycler was programmed as:

25 °C/10 min.

42 °C/30 min.

85 °C/5 min.

4 °C/hold.

#### 2.4.3. Real-Time Quantitative PCR Assays

The table below presents the recommended conditions and component volumes.

1. Reaction conditions

**Component**

**20 μL Reaction**

**Final Conc.**
qPCR Master (SYBR)10.0 μL1XROX Dye (50X) * (optional)0.4 μL1X10 μM Forward Primer0.2~2.0 μL0.1~1.0 μM10 μM Reverse Primer0.2~2.0 μL0.1~1.0 μMTemplate DNAVariable≤500 ng/reactionWater, RNase-Freeup to 20 μLNA

2. PCR conditions

**Step**

**Temp (°C)**

**Time**

**Cycle**
Initial Denaturation955 min1Denature9510~30 s30~40Anneal55~6810~60 s1Melting Curve Analysis65~952~5 s/stepCycle

3. In this study, we used four primers to detect the effect of ZnO NPs on gene expression. Primers were purchased from Bioneer/South Korea and applied in this work; the table below illustrates the primers and the detected target genes in Klebsiella spp. isolates:

**No.**

**Target Gene**

**Primer**

**Oligo Sequence (5′-3′)**

**Product Size (bp)**

**Ref.**
1
*magA*
magA-FGGT GCT CTT TAC ATC ATT GC1283(Turton et al., 2010)
magA-RGCA ATG GCC ATT TGC GTT TGC GTT AG2
*k2A*
k2A-FCAACCATGGTGGTCGATTAG543(Rivero et al., 2010) (Doud et al., 2009)k2A-RTGGTAGCCATATCCCTTTGG3
*rmpA*
rmpA-FACT GGG CTA CCT CTG CTT CA536(Nadasy et al., 2007) (Turton et al., 2010)rmpA-RCTT GCA TGA GCC ATC TTT CA4
*kfu*
kfu-FGAAGTGACGCTGTTTCTGGC797(Yu et al., 2008)kfu-RTTTCGTGTGGCCAGTGACTC

## 3. Results and Discussion

### 3.1. Antibiotic Resistance of K. pneumoniae Isolates

Table 1 shows the 18 different antibiotics that the 30 isolates of *K. pneumoniae* are noticed to be susceptible to. It was found that one hundred percent of *K. pneumoniae* isolates showed high resistance to cephalosporin drugs, whereas ampicillin, amoxicillin + clavulanic acid, and piperacillin had resistance rates of 100%, 97%, and 75%, respectively.

The excessive use of antibiotics in healthcare places, particularly with patients receiving many medicines, may result in specific mutations in bacteria and they may become multidrug resistant [15]. In order to avoid these issues, the usage of antibiotics in hospitals must be tracked and controlled. The plasmids that contain resistance genes have a significant function in spreading the multidrug resistance feature among bacteria in addition to mutation [16]. 

### 3.2. Impact of ZnO NPs on Capsular Gene Expression

The expression levels of the *magA*, *k2A*, *kfu*, and *rmpA* genes in *Klebsiella pneumoniae* isolates were examined using the RTq-PCR method under the influence of various ZnO nanoparticle concentrations. The findings demonstrate that in isolates treated with 0.25, 0.50, and 0.75 mM of ZnO nanoparticles, the transcript levels of the examined genes were reduced. 

#### 3.2.1. Effect of ZnO NPs on the Rate of *magA* Gene Expression

**Figure 1:**
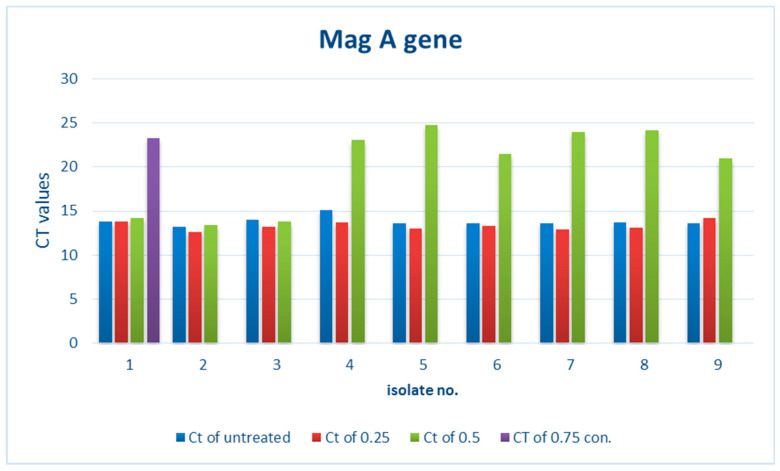
*magA* gene expression in *Klebsiella pneumoniae* isolates treated with different concentrations of ZnO NPs.

**Figure 2:**
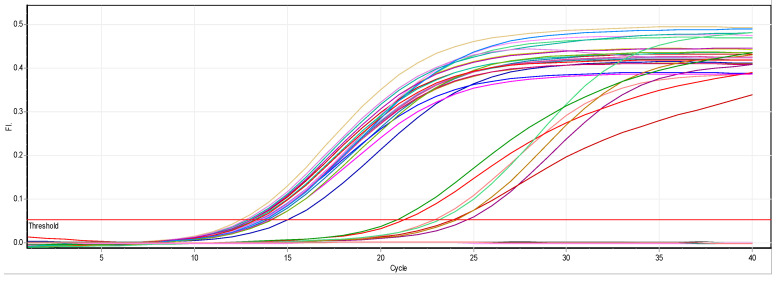
Real-time PCR results of the *magA* gene.

There is a noticeable effect on the gene expression levels of *magA* in *Klebsiella pneumoniae* isolates, as shown in Table 2 and Figure 1 and Figure 2. Colors in Table 2 represent the curves colors in the Figure 2. At a concentration of 0.25 mM, there was a slight change in the level of expression, and we noticed a reduction in the level of gene expression at a concentration of 0.50 mM. This decrease was higher at a concentration of 0.75 mM and led to a complete stop.

#### 3.2.2. Effect of ZnO NPs on the Rate of *k2A* Gene Expression

**Figure 3:**
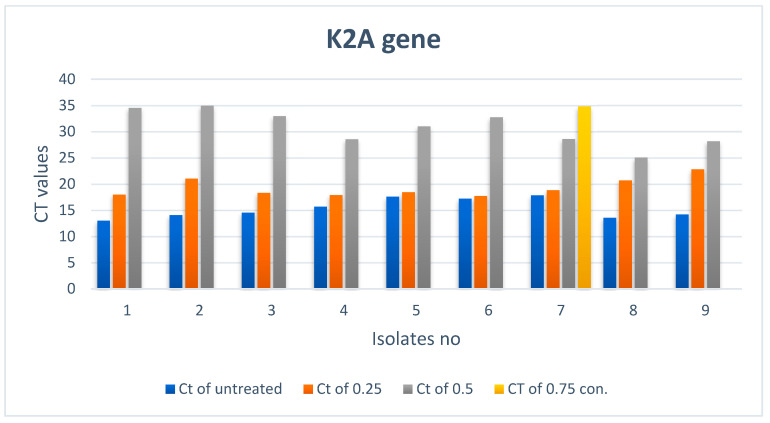
*k2A* gene expression in *Klebsiella pneumoniae* isolates treated with different concentrations of ZnO NPs.

**Figure 4:**
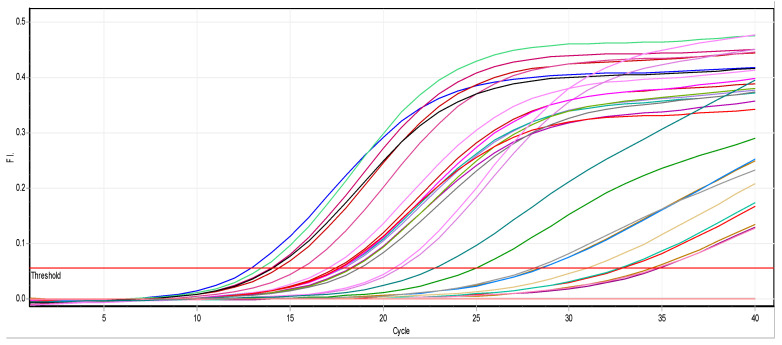
Real-time PCR results of the *k2A* gene.

There is a noticeable effect on the gene expression levels of *k2A* in *Klebsiella pneumoniae* isolates, as shown in Table 3 and Figure 3 and Figure 4. Colors in the Table 3 represent the curves colors in Figure 4. At a concentration of 0.25 mM, there was a slight change in the level of expression, and at a concentration of 0.50 mM, the level of gene expression began to decrease. However, it completely stopped at a concentration of 0.75 mM.

#### 3.2.3. Effect of ZnO NPs on the Rate of *kfu* Gene Expression

**Figure 5:**
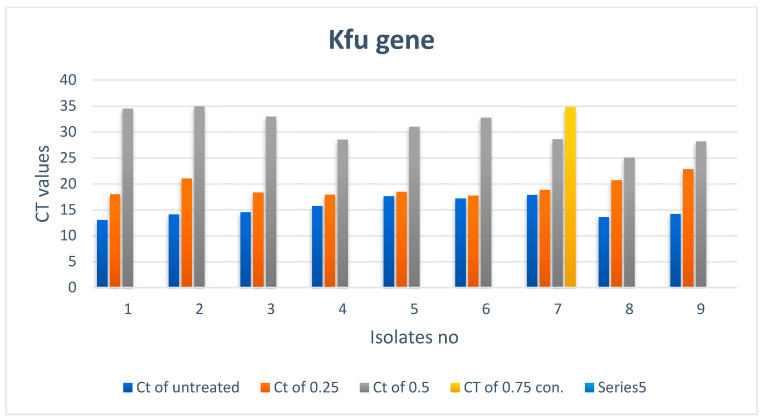
*kfu* gene expression in *Klebsiella pneumoniae* isolates treated with different concentrations of ZnO nanoparticles.

**Figure 6:**
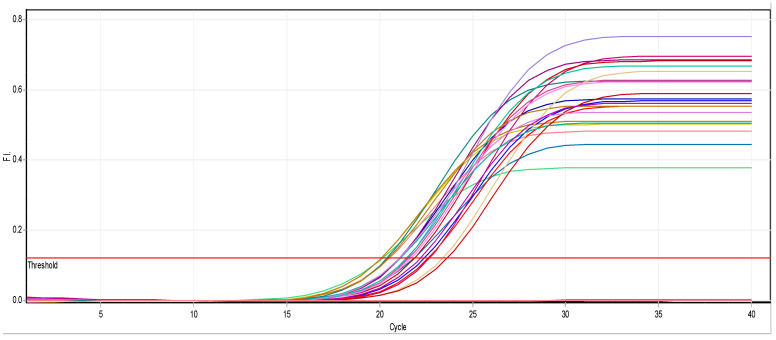
Real-time PCR results of the *kfu* gene.

There is a noticeable effect on the *kfu* gene expression levels in *Klebsiella pneumoniae* isolates, as shown in Table 4 and Figure 5 and Figure 6. Colors in the Table 4 represent the curves colors in Figure 6. At a concentration of 0.25 mM, there was a slight change in the level of expression, and it was noticed that the level of gene expression began to decrease at a concentration of 0.50 mM. Finally, the gene expression completely stopped at a concentration of 0.75 Mm.

#### 3.2.4. Effect of Zinc Oxide on the Rate of the Gene Expression of *rmpA*

**Figure 7:**
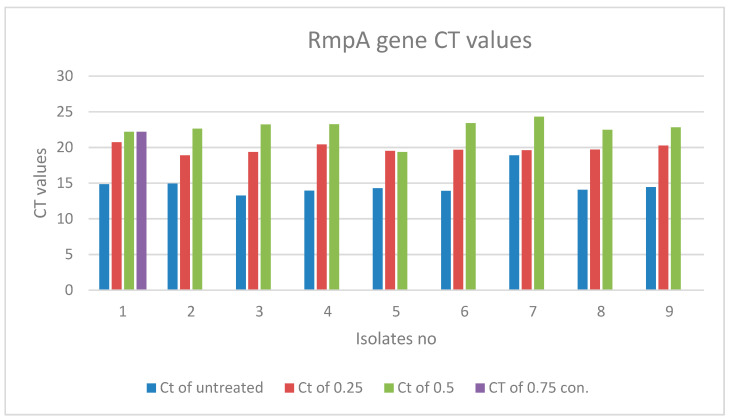
*rmpA* gene expression in *Klebsiella pneumoniae* isolates treated with different concentrations of ZnO NPs.

**Figure 8:**
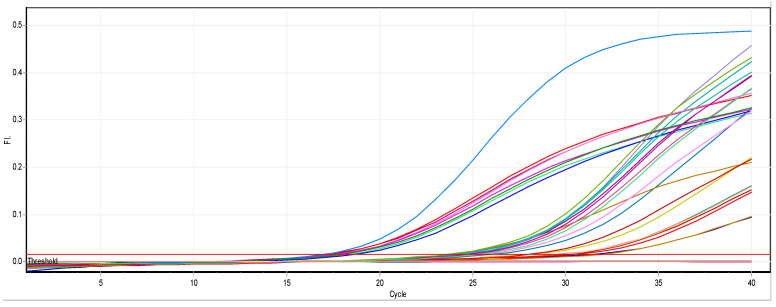
Real-time PCR results of the *rmpA* gene.

There is a noticeable effect on the gene expression levels of *rmpA* in Klebsiella pneumoniae isolates, as shown in Table 5 and Figure 7 and Figure 8. Colors in the Table 5 represent the curves colors in Figure 8. At a concentration of 0.25 mM, there was a slight change in the level of expression. Nevertheless, it is noticed that at a concentration of 0.50 mM, the level of gene expression began to decrease, and finally completely stopped at a concentration of 0.75 mM.

## 4. Discussion

New, harmful, antibiotic-resistant bacterial strains have emerged because of the indiscriminate utilization of antibiotics in contemporary culture. *K. pneumoniae* is a significant human pathogen that was recently linked to outbreaks in hospitals. Extended-spectrum b-lactamase (ESBL)-producing *K. pneumoniae* has been raised as a main and widespread issue because of the usage of prolonged-spectrum cephalosporins [17,18]. Here, new evidence states that an effective bacterial antibiotic in the form of NPs has been presented [19]. This could suggest a solution to the crucial issue of antibiotic resistance while lowering the danger of infections and their associated consequences; these have a serious impact on weak hospital patients. The development of novel and highly powerful bactericidal agents is motivated by the emergence of antibiotic-resistant microorganisms [20]. The current investigation’s findings point out three potential antimicrobial mechanisms for ZnO NP suspensions. (1) A physical mechanism that involves direct attachment to bacteria’s cell walls; (2) a biological mechanism that involves interaction with the components of the cell membrane; and (3) a chemical mechanism that involves the production of active species [21]. The size and concentration of ZnO play an important role in antibacterial activity. H_2_O_2_ production mainly depends on the surface area of nano ZnO; larger surface areas and higher concentrations of smaller particles may provide additional antibacterial activity [8]. This is in good agreement with a study on *Escherichia coli* and *Pseudomonas aeruginosa*, which were also sensitive to ZnO NPs, observing that NPs ranging from 14 to 25 nm inhibit them. Moreover, ZnO nanoparticles of a size of 22 nm were used against *Klebsiella pneumonia* and good results were obtained [7].

In general, the main findings imply that ZnO NPs could be employed externally to prevent bacterial infections from spreading. However, enhancing the ZnO NP concentrations slows the reproduction of *K. pneumoniae*, according to the MIC test and variation in the conventional growth curve. Gram-negative bacteria are found to have a higher negative charge on their cell surfaces than Gram-positive bacteria [22]. In some cases, membrane permeability is the initial stage of bacteria’s resistance to an antibiotic [23]. Marsalek measured the zeta potential of ZnO, where it was +35 mV at a pH of 7. This high positive value works to attract the bacteria due to the difference in charge [24]. Hence, there is a strong antibacterial impact when ZnO NPs exhibit their full potential by directly interfering with the degradation of the LPS membrane. The bacterial attachment is affected by the surface charge and specific surface hydrophobicity; these properties are assumed to be effective on bacterium surface adherence. The impact of the LPS membrane in adherence may be implicated in non-limited physicochemical interactions [25]. By subjecting the bacteria to oxidative stress and preventing bacterial development, ZnO NPs reduce the production of catalase, which is an antioxidant enzyme that shields the bacterium from oxidative stress [26].

On the other hand, several studies indicated ROS formation as the main mechanism for ZnO NP antibacterial activity [27]. The reactive species include superoxide anion (O_2_), hydrogen peroxide (H_2_O_2_), and hydroxide (OH−). The toxicity of these species involves the destruction of cellular components, such as lipids, DNA, and proteins, as a result of their internalization into the bacterial cell membrane. However, the role of ROS in antimicrobial actions has become an issue of debate among researchers in this field [28]. More studies should be done to correlate the role of ROS with ZnO NPs, but our study focuses on the effect of ZnO NPs on the gene expression of the genes responsible for capsular structures.

## 5. Conclusions

The zinc oxide nanoparticles were found to have antimicrobial activity against *Klebsiella pneumoniae.* This provides the potential to use this material in therapeutic material (antimicrobial agent) production. The ZnO NPs reduced the gained resistance incidence among pathogens from the misapplication and/or overuse of antibiotics. After investigating different concentrations of the NPs, 0.75 mM was found to be very effective in terminating the bacteria genes. 

## Figures and Tables

**Table 1:** Antibiotic resistance of *K. pneumoniae* isolates.

Antibiotic	Resistant%	Intermediate%	Sensitive%
Piperacillin	74%	4%	22%
Cefazolin	84%	0%	16%
Cefoxitin	55%	0%	45%
Ceftazidime	63%	10%	27%
Ceftriaxone	100%	0%	0%
Cefepime	40%	0%	60%
Imipenem	21%	6%	73%
Amikacin	10%	10%	80%
Gentamicin	35%	5%	60%
Ciprofloxacine	35%	0%	65%
Levofloxacin	34%	0%	66%
Tigecyclin	0%	0%	100%
Nitrofurantoin	16%	42%	42%
Trimethoprim	75%	0%	25%
Ampicillin	100%	0%	0%
Amoxicillin	97%	0%	3%
Cephalothin	100%	0%	0%
Cephradine	100%	0%	0%

**Table 2:** *magA* gene expression in *Klebsiella pneumoniae* isolates treated with different concentrations of ZnO NPs *.

Treated Bacteria Isolates with Different con.							Untreated	
No	Ct of con3 (0.75)	Color	Ct of con2 (0.5)	Color	Ct of con1 (0.25)	Color	Ct	Color
1	23.27	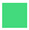	14.24	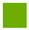	13.78	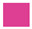	13.79	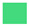
2	Not expressed	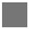	13.43	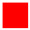	12.63	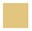	13.2	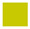
3	Not expressed	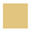	13.84	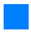	13.2	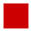	13.99	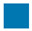
4	Not expressed	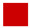	23.01	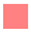	13.75	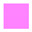	15.13	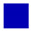
5	Not expressed	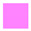	24.75	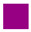	13.04	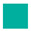	13.65	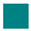
6	Not expressed	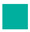	21.42	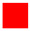	13.32	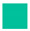	13.61	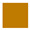
7	Not expressed	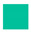	23.96	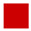	12.9	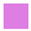	13.64	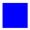
8	Not expressed	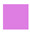	24.15	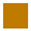	13.14	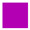	13.69	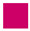
9	Not expressed	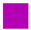	20.99	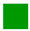	14.18	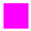	13.6	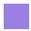

* CT: cycle of threshold; con: concentration in mM.

**Table 3:** *k2A* gene expression in *Klebsiella pneumoniae* isolates treated with different concentrations of ZnO NPs *.

Treated Bacteria Isolates with Different con.							Untreated	
	Ct of con3 (0.75)	Color	Ct of con2 (0.5)	Color	Ct of con1 (0.25)	Color	Ct	Color
1	Not expressed	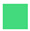	34.51	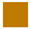	17.98	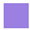	13	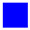
2	Not expressed	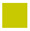	34.95	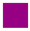	21.04	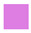	14.09	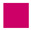
3	Not expressed	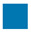	32.97	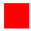	18.33	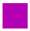	14.53	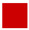
4	Not expressed	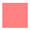	28.53	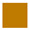	17.88	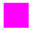	15.7	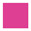
5	Not expressed	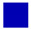	31.01	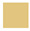	18.46	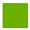	17.58	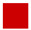
6	Not expressed	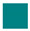	32.76	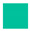	17.72	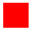	17.19	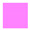
7	34.8	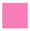	28.59	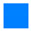	18.82	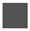	17.86	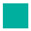
8	Not expressed	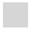	25.04	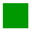	20.7	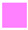	13.58	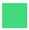
9	Not expressed	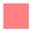	28.15	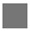	22.83	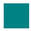	14.19	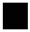

* CT: cycle of threshold; con: concentration in mM.

**Table 4:** *kfu* gene expression in *Klebsiella pneumoniae* isolates treated with different concentrations of ZnO NPs *.

Treated Bacteria Isolates with Different con.							Untreated	
No	Ct of con3 (0.75)	Color	Ct of con2 (0.5)	Color	Ct of con1 (0.25)	Color	Ct	Color
1	Not expressed	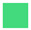	34.51	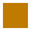	17.98	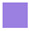	13	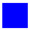
2	Not expressed	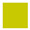	34.95	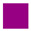	21.04	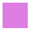	14.09	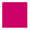
3	Not expressed	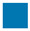	32.97	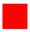	18.33	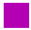	14.53	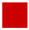
4	Not expressed	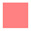	28.53	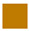	17.88	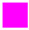	15.7	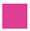
5	Not expressed	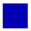	31.01	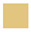	18.46	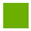	17.58	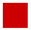
6	Not expressed	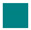	32.76	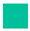	17.72	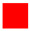	17.19	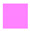
7	34.8	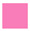	28.59	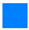	18.82	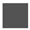	17.86	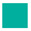
8	Not expressed	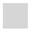	25.04	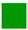	20.7	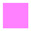	13.58	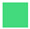
9	Not expressed	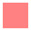	28.15	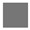	22.83	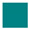	14.19	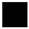

* CT: cycle of threshold; con: concentration in Mm.

**Table 5:** *rmpA* gene expression in *Klebsiella pneumoniae* isolates treated with different concentrations of ZnO NPs *.

Treated Bacteria Isolates with Different con.							Untreated	
No	Ct of con3 (0.75)	Color	Ct of con2 (0.5)	Color	Ct of con1 (0.25)	Color	Ct	Color
1	22.19	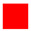	22.18	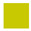	20.73	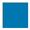	14.83	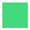
2	Not expressed	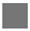	22.63	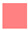	18.9	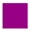	14.93	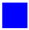
3	Not expressed	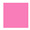	23.22	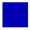	19.35	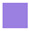	13.24	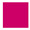
4	Not expressed	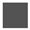	23.24	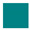	20.4	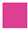	13.95	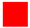
5	Not expressed	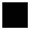	19.36	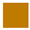	19.51	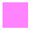	14.28	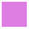
6	Not expressed	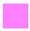	23.39	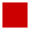	19.65	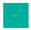	13.89	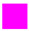
7	Not expressed	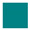	24.29	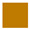	19.59	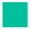	18.88	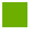
8	Not expressed	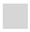	22.48	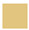	19.68	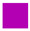	14.06	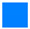
9	Not expressed	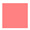	22.82	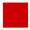	20.25	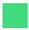	14.44	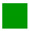

* CT: cycle of threshold; con: concentration in Mm.

## References

[1] Chiu S., Wu T., Chuang Y., Lin J., Fung C., Lu P., Wang J., Wang L., Siu K., Yeh K. (2013). National surveillance study on carbapenem Non-susceptible Klebsiella pneumoniae in taiwan: The emergence and rapid dissemination of kpc-2 carbapenemase. PLoS ONE.

[2] Aher T., Roy A., Kumar P. (2012). Molecular detection of virulence genes associated with pathogenicity of *Klebsiella* spp. isolated from the respiratory tract of apparently healthy as well as sick goats. J. Vet. Med..

[3] Dubey D., Raza F., Sawhney A., Pandey A. (2013). *Klebsiella pneumoniae* renal abscess syndrome: A rare case with metastatic involvement of lungs, eye, and brain. Case Rep. Infect. Dis..

[4] Fang C., Lin Y., Lin J., Chen T., Yeh K., Chang F., Chuang H., Wu H., Tseng C., Siu L. (2012). *Klebsiella pneumoniae* in Gastrointestinal Tract and Pyogenic Liver Abscess. Emerg. Infect. Dis..

[5] Doud M., Zeppegno R., Molina E., Miller N., Balachandar D., Schneper L., Poppiti R., Mathee K. (2009). A *k2A*-positive *Klebsiella pneumoniae* causes liver and brain abscess in a Saint Kitt’s man. Int. J. Med. Sci..

[6] Turton J., Perry C., Elgohari S., Hampton C. (2010). PCR characterization and typing of *Klebsiella pneumoniae* using capsular type-specific, variable number tandem repeat and virulence gene targets. J. Med. Microbiol..

[7] Azam A., Ahmed A., Oves M., Khan M., Memic A. (2012). Size-dependent antimicrobial properties of CuO nanoparticles against Gram-positive and-negative bacterial strains. Int. J. Nanomed..

[8] Padmavathy N., Vijayaraghavan R. (2008). Enhanced Bioactivity of ZnO Nanoparticles an Antimicrobial Study. Sci. Technol. Adv. Mater..

[9] Jiang J., Pi J., Cai J. (2018). The Advancing of Zinc Oxide Nanoparticles for Biomedical Application. Bioinorg. Chem. Appl..

[10] Ravishankar Rai V., Jamuna Bai A., Méndez-Vilas A. (2011). Nanoparticles and Their Potential Application as Antimicrobials. Science against Microbial Pathogens: Communicating Current Research and Technological Advances.

[11] Brayner R., Ferrari-Iliou R., Brivois N., Djediat S., Benedetti M.F., Fievet F. (2006). Toxicological impact studies based on *Escherichia coli* bacteria in ultrafine ZnO nanoparticles colloidal medium. Nano Lett..

[12] Huang Y., Liao H., Wu C., Peng H. (2009). MrkF is a component of type 3 fimbriae in *Klebsiella pneumoniae*. J. Res. Microbiol..

[13] Kudaer N.B., Yousif E., Risan M.H., Kadhom M. (2022). Preparation of ZnO Nanoparticles by the Chemical Precipitation Method. J. Serambi Eng..

[14] Holt J.G., Krieg N.R. (1994). Bergey’s Manual of Determinative Bacteriology.

[15] Hickling T.P., Clark H., Malhotra R., Sim R.B. (2004). Collectins and theirrole in lung immunity. J. Leukoc. Biol..

[16] Tullus K. (1987). Fecal colonization with P-fimbriated *Escherichia coli* in newborn children and relation to development of extraintestinal *E. coli* infections. Acta Paediatr. Scand..

[17] Stock I., Wiedemann B. (2001). Natural antibiotic susceptibility of *Klebsiella pneumoniae*, *K. oxytoca*, *K. planticola*, *K. ornithinolytica* and *K. terrigena* strains. J. Med. Microbiol..

[18] Schurr J.R., Young E., Byrne P. (2005). Central role of toll-like receptor 4 signaling and host defense in experimental pneumonia caused by Gram-negative bacteria. Infect. Immun..

[19] Huh A.J., Kwon Y.J. (2011). Nanoantibiotics: A new paradigm for treating infectious diseases using nanomaterials in the antibiotics resistant era. J. Control Release.

[20] Mohammed S.I., Chandrasekaran N., Mukherjee A. (2010). Studies on effect of TiO2 nanoparticles on growth and membrane permeability of *Escherichia coli*, *Pseudomonas aeruginosa*, and *Bacillus subtilis*. Curr. Nanosci..

[21] Lingling Z., Yunhong J., Yulon D. (2007). Investigation into the antibacterial behaviour of suspensions of ZnO nanoparticles (ZnO nanofluids). J. Nanopart. Res..

[22] Chung Y.C., Su Y.P., Chen C.C. (2004). Relationship between antibacterial activity of chitosan and surface characteristics of cell wall. Acta Pharmacol. Sin..

[23] Berry V., Gole A., Kundu S. (2005). Deposition of CTABterminated nanorods on bacteria to form highly conducting hybrid systems. J. Am. Chem. Soc..

[24] Marsalek R. (2014). Particle size and zeta potential of ZnO. APCBEE Proc..

[25] Klotz S., Doyle R., Rosenberg M. (1990). Role of hydrophobic interactions in microbial adhesion to plastics used in medical devices. Microbial Cell Surface Hydrophobicity.

[26] Keisari Y., Wang H., Mesika A. (2001). Surfactant protein D-coated *Klebsiella pneumoniae* stimulates cytokine production in mononuclear phagocytes. J. Leukoc. Biol..

[27] Jalal R., Goharshadi E., Abareshi M., Moosavi M., Yousefi A., Nancarrow P. (2010). ZnO nanofluids: Green synthesis, characterization, and antibacterial activity. Mater. Chem. Phys..

[28] Raghupathi K., Koodali R., Manna A. (2011). Size-Dependent Bacterial Growth Inhibition and Mechanism of Antibacterial Activity of Zinc Oxide Nanoparticles. Langmuir.

